# Manipulating the Biosynthesis of Bioactive Compound Alkaloids for Next-Generation Metabolic Engineering in Opium Poppy Using CRISPR-Cas 9 Genome Editing Technology

**DOI:** 10.1038/srep30910

**Published:** 2016-08-03

**Authors:** Yagiz Alagoz, Tugba Gurkok, Baohong Zhang, Turgay Unver

**Affiliations:** 1 Department of Biology, Faculty of Science, Çankırı Karatekin University, Çankırı18100, Turkey; 2 Hawkesbury Institute for the Environment, Western Sydney University, Richmond, New South Wales 2753, Australia; 3 Department of Biology, East Carolina University, Greenville, NC 27858, USA; 4 Izmir International Biomedicine and Genome Institute (iBG-izmir), Dokuz Eylul University, Balcova 35340 Izmir, Turkey

## Abstract

Clustered regularly interspaced short palindromic repeats (CRISPR)/CRISPR-associated9 (Cas9) endonuclease system is a powerful RNA-guided genome editing tool. CRISPR/Cas9 has been well studied in model plant species for targeted genome editing. However, few studies have been reported on plant species without whole genome sequence information. Currently, no study has been performed to manipulate metabolic pathways using CRISPR/Cas9. In this study, the type II CRISPR/SpCas9 system was used to knock out, via nonhomologous end-joining genome repair, the *4*′*OMT2* in opium poppy (*Papaver somniferum* L.), a gene which regulates the biosythesis of benzylisoquinoline alkaloids (BIAs). For sgRNA transcription, viral-based TRV and synthetic binary plasmids were designed and delivered into plant cells with a Cas9 encoding-synthetic vector by *Agrobacterium*-mediated transformation. InDels formed by CRISPR/Cas9 were detected by sequence analysis. Our results showed that the biosynthesis of BIAs (e.g. morphine, thebaine) was significantly reduced in the transgenic plants suggesting that *4*′*OMT2* was efficiently knocked-out by our CRISPR-Cas9 genome editing approach. In addition, a novel uncharacterized alkaloid was observed only in CRISPR/Cas9 edited plants. Thus, the applicabilitiy of the CRISPR/Cas9 system was demonstrated for the first time for medicinal aromatic plants by sgRNAs transcribed from both synthetic and viral vectors to regulate BIA metabolism and biosynthesis.

Plants provide many nutrients and metabolites that are either directly assimilated by humans or used as raw material in different industries. Recently, new plant breeding technologies have been employed in order to obtain a multitude of beneficial traits like increased metabolite and nutrient production and resistance to harsh stresses.

Protein based editing tools, including zinc-finger nucleases (ZFNs) and transciption activator-like endunucleases (TALENs), have been powerful tools for manipulating genomes at the transcriptional level in the past few years^1^. ZFN and TALEN technologies employ a protein-based endonuclease called Fok-I to knock-out the gene of interest by introducing double-strand breaks (DSBs) in the target DNA^2^. Targeting desired sequences in the genome can be troublesome with these systems as they require designing two different protein hybrids that can recognize rarely occurring regions flanking the target site^3^,^4^. The efficiencies of these systems are challenged by the presence of those DNA sequences along with the ability of both proteins to orient correctly and dimerize with the provided spacer length^5^,^6^. Some of those difficulties were overcome with the discovery of the CRISPR/Cas9 system that does not require protein dimerization^7^. The system promotes gene editing by utilizing well-designed guide RNAs that recognizes a 3 base-pair PAM sequence downstream of the target site^7^,^8^,^9^,^10^,^11^. Since the first implementation of CRISPR system in plants, it has been applied by many researchers in several plant species including *Arabidopsis thaliana*^12^*, Brassica oleracea*^13^, *Citrus sinensis*^14^, *Cucumis sativus*^15^, *Glycine max*^16^, *Hordeum vulgare*^13^, *Lactuva sativa*^17^, *Marchantia polymorpha*^18^*, Medicago truncatula*^19^*, Nicotiana attenuate*^17^, *Nicotiana benthamiana*^12^,^20^*, Nicotiana tabacum*^21^*, Oriza sativa*^22^*, Populus tomentosa*^23^, *Populus tremula*^24^, *Solanum lycopersicum*^25^, *Solanum tuberosum*^26^, *Sorghum bicolor*^27^*, Triticum aestivum*^28^, and *Zea mays*^29^.

*P. somniferum* (2n = 22), from the family of *Papaveraceae*, is one of the two opium poppy species known to biosynthesize morphine^30^,^31^. Despite of its importance as an oil plant, opium poppy also produces many benzylisoquinoline alkaloids (BIAs) used in biomedicine, like antitussive codeine and noscapine, anticancer noscapine, antispasmodic papaverine and analgesic morphine. BIA biosynthesis starts with the condensation of dopamine and the 4-hydroxyphenylacetaldehyde (4-HPAA) to yield (S)-norcoclaurine^32^. After a series of methylation and hydroxylation steps, (S)-norcoclaurine is converted to central intermediate S-reticuline^33^,^34^. This step is catalysed by 3′-hydroxy-N-methylcoclaurine 4′-O-methyltransferase (4′OMT)^35^ (Fig. 1). Several end products such as morphine, noscapine and papaverine are derived from S-reticuline via different BIA pathways. The epimerization of (S)- reticuline to (R)-reticuline results in the production of morphine (morphinian)^36^. Moreover, the conversion of (S)-reticuline to (S)-scoulerine is catalyzed by Berberine Bridge Enzyme (BBE) and this is the first committed step in the biosynthesis of sanguinarine (benzophenanthridine) and noscapine (phthalideisoquinoline) alkaloids^37^.

Recently, it has been reported that BIA production in opium poppy can be manipulated by altering the expression of some particular genes in the BIA pathway. Over-expression and TRV-mediated gene silencing studies in opium poppy revealed that the amount of alkaloid biosynthesis could be manipulated in a tissue-specific manner^38^,^39^,^40^,^41^. Both the over-expression and the silencing of (*R, S*)*-reticuline 7-O-methyltransferase* (*7OMT*) and *3*′*-hydroxyl-N-methylcoclaurine 4*′*-O-methyltransferase* (*4*′*OMT2*) genes revealed their regulatory roles in BIA production in different tissues^42^. Nevertheless, the roles of these genes cannot be completely understood using the aforementioned strategies. In particular, the previous approaches impacted gene expression at the post-transcriptional level. This results in a significant decrease in gene expression but does not abolish the gene function. This is important, as the biosynthetic pathways do not cease and the production of metabolites continues, which could mask the overall phenotypes regulated by the targeted genes. Therefore the application of gene knock-out strategies, like CRISPR/Cas9, can help address these challenges and increase our understanding of the roles of the genes of interest.

Synthetic vectors are usually used to express CRISPR system components like sgRNAs and Cas9 endonuclease in plant and animal systems. However, synthetic vectors can be expressed in the transfected or transformed cells only and not the neighbouring cells. Additionally, viral vectors are not suitable for nucleic acid constructs like the one encoding SpCas9 (*Streptococcus pyogenes* Cas9)^21^. The efficiency of the CRISPR/Cas9 system could be increased by using viral vector backbones for the expression of guide RNAs in plant systems. In this study, we used the CRISPR/Cas9 system to knock out *4*′*OMT* (isoform-2) which is a critical regulatory gene involved in the biosynthesis of some bioactive compounds produced in the medicinal aromatic plant, opium poppy (*Papaver somniferum* L.) using both synthetic and viral-based delivery systems.

## Materials and Methods

### Plant materials

*P. somniferum* (cv. *Ofis*-*95*) seeds were obtained from Toprak Mahsulleri Ofisi (TMO, Ankara, Turkey) and grown in a mixture of 50% perlite and 50% peat in an environment of 24/20 °C with 16/8 hours light/dark and 60% humidity in a plant growth cabinet.

### Vectors

Vectors of 46965, 46966, 46968, and 48002 were purchased from Addgene (www.addgene.org, MA, US) while pTRV2::AtU6p::sgRNA_4OMT2 (Supplementary Fig. S1), pICH47751::AtU6p::sgRNA_4OMT2 (Supplementary Fig. S2), and pTRV1 were synthesized in our lab. Plasmid isolations were carried out using GeneJET Plasmid Miniprep Kit (Thermo Scientific, Massachusetts, US) according to the manufacturer’s protocol. The sgRNA delivery constructs were specifically designed to target and edit the *4*′*OMT2* sequence (GenBank ID: AY217334.1) (Supplementary Fig. S3).

### Construction of synthetic vectors

*4*′*OMT2* targeting sgRNA was synthesized by classical PCR with 4OMT2sgRNA_1F and 4OMT2sgRNA_1R primers (Table S1) using approximately 100 ng of plasmid 46966 as a template. Then, PCR products were purified using High Pure PCR Product Purification Kit (Roche, Manheim, Germany). The AtU6p promotor and synthesized sgRNA were assembled by MoClo method^43^. The single-step reaction was done by the proper combination of 40 fmoles of each vector (46968: an AtU6p encoding L0 plasmid, plasmid 48002: empty backbone plasmid used as MoClo L1 destination vector) and 100 ng purified sgRNA_4OMT2 amplicon with 2.5 Weiss units of Bsa I (New England Biolabs, MA, US) along with 200 CEU of T4 Ligase (Thermo Scientific, Massachusetts, US), 1.5 μL of 10 × T4 ligase buffer, and 1.5 μL 10 × BSA to the final volume of 15 μL. The reaction mixture was incubated in a thermal-cycler for 1 min at 37 °C, followed by 30 cycles alternating between 3 min at 37 °C and 4 min at 16 °C, and one cycle of 5 min 50 °C and 5 min at 80 °C, respectively. The end-product was chemically transferred into competent *E. coli* (strain DH10B) cells by heat-shock for blue-white colony screening.

### Construction of viral vectors

sgRNApaIF and sgRNApaIR primers were used to link Apa I recognition sites to the flanking sites of AtU6p::sgRNA_4OMT2 insert by PCR (Table S1). The PCR product was purified using High Pure PCR Product Purification Kit (Roche, Manheim, Germany) and was then ligated into pGEM^®^-T (Promega, Wisconsin, US), T-A cloning vector using T4 DNA ligase. Ligation mixture was transfected to chemically competent *E. coli* cells by heat-shock and positive clones were then confirmed by colony-PCR using sgRNApaIF and sgRNApaIR primers. Plasmids were further verified with Sanger dideoxy sequencing (RefGen, Ankara, Turkey). Verified inserts were digested and ligated into pTRV expression vectors for in-planta expression using T4 DNA ligase. Ligation products were transfected into chemically competent *E. coli* cells and grown in an appropriate medium for colony selection with PCR utilizing sgRNApaIF and sgRNApaIR primers (Table S1). Chemically competent cells were prepared with RbCl buffers according to a previous report^44^.

### Agro-infiltration of the constructs into plant cells

**Figure 1:**
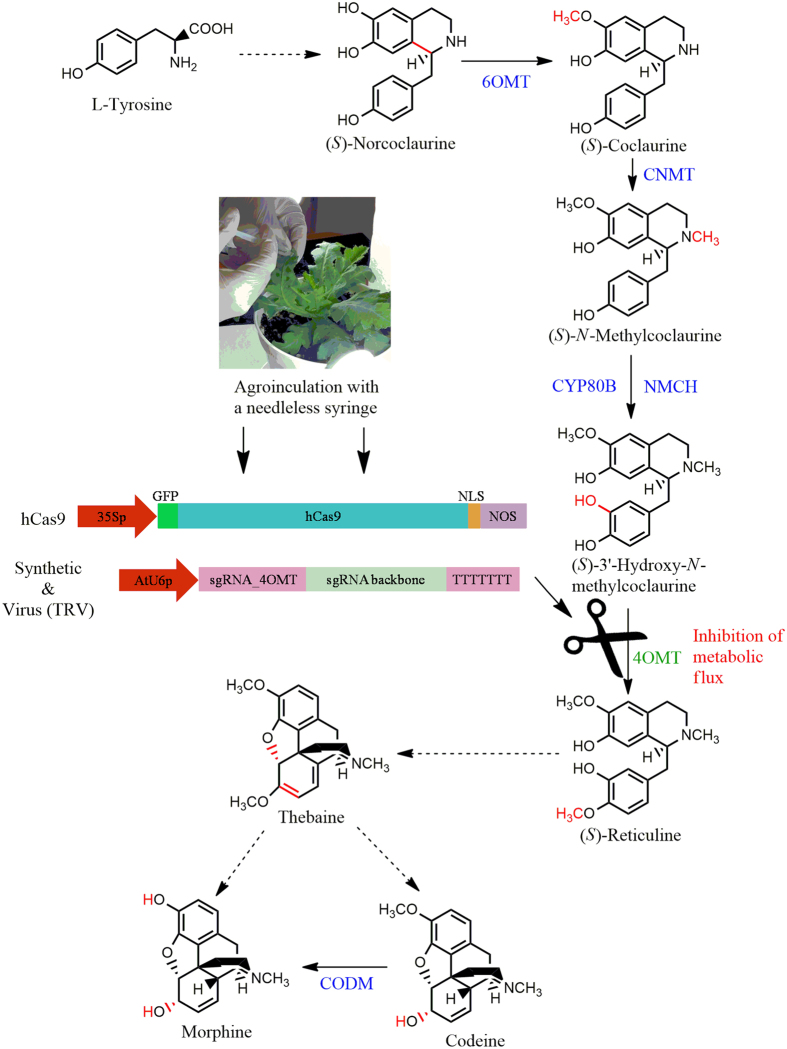
Morphine, thebaine and codeine biosynthesis in opium poppy is catalyzed by several essential enzymes including *4*′*OMT2*. By delivering CRISPR/Cas9 system components of sgRNA and Cas9 inside plant tissues with agroinoculation, it’s possible to knock-out the desired gene segment to manipulate BIA production flux for engineered biosynthesis. This figure shows the target for CRISPR/Cas9.

Plasmids, containing 35S::h*Cas9* and AtU6p:sgRNA_4OMT2 inserts were transferred into the electro-competent *A. tumefaciens* (strain EHA105 and GV3101) cells by using an electroporator (Invitrogen, Neon Transfection System, Massachusetts, US). Alternatively, chemically competent cells were prepared by using CaCl_2_ according to Holsters’s protocol^45^. Plasmids were harvested from bacteria grown overnight(OD_600_: 2-2.5) and re-suspended in a final concentration of OD_600_: 0.8 in an induction buffer containing 10 mM MgCl_2_, with 100 μM acetosyringone and 1 mM MES at pH 5.6 46. The prepared mixture was incubated overnight at room temperature and infiltrated into leaves by a needleless syringe (Fig. 1). Inoculations of the synthetic plasmids were performed using 1:1 ratio of mixed bacteria containing each sgRNA and hCas9 expressor plasmids. Twenty 8–10 weeks old plants were co-infiltrated for each treatment with vectors of pK7WGF2::hCas9 (Addgene plasmid #46965) and pICH47751:: AtU6p::sgRNA_4OMT2. For TRV-mediated CRISPR/Cas9 implementations, TRV1, TRV2 (pTRV2::AtU6p::sgRNA_4OMT2) and hCas9 encoding pK7WGF2::hCas9 (Addgene #46965) plasmids were infiltrated in 1:1:1 ratio to precisely maintain the expression of Cas9 inside plant tissues. Two days after the initial infiltration, this process was repeated one more time to enhance sgRNA/Cas9 effectivity.

### gDNA isolation from sgRNA_Cas9 treated tissues

After 4 days of agro-infiltration, leaves were harvested for gDNA isolation. gDNA from both control group and treated plants were extracted by using standardized CTAB & sucrose plant DNA extraction protocol. 100 mg of frozen leaves were mechanically homogenized using TissueLyser II system (Qiagen, Hilden, Germany) and each sample was treated with RNase A (Qiagen). Isolated gDNA samples were enriched by enzymatic digestion assay performed by digesting gDNAs with Age I (Jena Biosciences, Jena, Germany) for *4*′*OMT2* targeted sequences.

### Analysis of InDels with DNA sequencing

100 ng of the enriched gDNA was used to clone the targeted regions of the *4*′*OMT2* gene for each experimental group. Targeted DNA segments were amplified with PCR by using High Fidelity DNA polymerases (Jena Biosciences, Jena, Germany). For the amplification of *4*′*OMT2* region, primers of 4OMT2_AgeIF and 4OMT2_AgeIR were used. In order to eliminate non-digested DNA, amplicons were further enriched by a second digestion assay and run on a 3% agarose gel for AFLP analyses. Identified DNA segments with potential insertion and deletions (InDels) were isolated from gel by using a GeneJET Gel Extraction Kit following the manufacturer’s protocol and then cloned into pGEM^®^-T vector by T4 DNA ligase reaction. The end product was transfected to *E. coli* DH5α cells for blue-white colony screening. After verifying positive colonies with M13 colony PCR, plasmids containing *4*′*OMT2* gene segments were isolated using a GeneJET Plasmid Miniprep Kit (Thermo Scientific, Massachusetts, US) as described in the manufacturer’s protocol and further analysed with Sanger dideoxy sequencing. 20-nt of the *4*′*OMT2* targeting segment in the sgRNA sequence was used for a search in the BLASTN program of NCBI. In particular, the seed sequence of sgRNA (N)_12,_ was examined to look for potential possible off-targets using our CRISPR/Cas9 system.

### Metabolite Analyses

To determine the products of the BIA pathway, the opium poppy leaves were collected after 4 days of agro-infiltration and dried at 28 °C for 2–3 days according to previous reports^41^,^47^. Then, 0.1 g of dried samples were soaked in methanol (HPLC grade, Merck, Darmstadt, Germany) at room temperature and incubated for 24 hours. Four biological replicates were used for each control and transgenic line. For separation, marc filtration was performed and the samples were evaporated. The extracts were dissolved to a concentration of 2,000 ppm and diluted to 1/200 ppm from stock. The extracts were filtered through 0.45 μm membranes for HPLC-TOF/MS analysis. Morphine, codeine, thebaine, papaverine, noscapine and laudanosine alkaloids were analysed using their respective standards. Alkaloids were quantified in an Agilent 1260 Infinity HPLC system (Agilent, Palo Alto, CA) coupled with Agilent 6210 TOF-MS detector and Agilent EC 250/4 Nucleosil 100-5 (HPLC Column, Nucleosil C18, 100A, 5um, 4 × 250 mm). The column temperature was adjusted to 35 °C and the injection volume was set to 10 μL. Mobile phases A and B are water 1 mg/L and acetonitrile (in 0.1% formic acid) respectively. The elution program was: 0–6 min, 40% B; 6–10 min, 50% B; 10–15 min, 90% B; 15-16 min, 90% B; 16–25 min, 40% B. The statistical analysis was performed using one-way ANOVA test.

## Results

### Knock-out of 4′OMT2 with CRISPR/Cas9 system

In order to examine the efficiency of sgRNA mediated CRISPR/Cas9 genome editing in opium poppy. We targeted the poppy *4*′*OMT2* gene by introducing double strand brakes (DSBs) and observed InDel mutations directed by Non-Homologous End Joining (NHEJ) repair mechanism. A 20-bp sequence in the 5′ region of the *4*′*OMT2* locus included a 3 bp protospacer adjacent motif (PAM) was selected for targeting by sgRNA transcribed from both synthetic and viral vectors. The *U6* promoter of *A. thaliana* (*AtU6p*) and sgRNA_4OMT2 (with 38.8% GC content) were assembled in a single cut-ligation MoClo L1 (Modular Cloning Level-1) reaction. The assembled sequence of AtU6p_sgRNA_4OMT2 was cloned into the ptv:00 (pTRV2) vector. These two cassettes were used as binary vectors with an hCas9 encoding plasmid (Addgene #46965) to transform the CRISPR/Cas9 system into plant cells. pTRV1 was also used during the transformation of the TRV-mediated CRISPR/Cas9 system for the effective expression of pTRV2 in plant tissues. Simultaneous expression of sgRNA and Cas9 in opium poppy resulted in the induction of frame-shift mutation in desired segments of the genome.

### Identification of mutated gene segments in 4′OMT2

**Figure 2:**
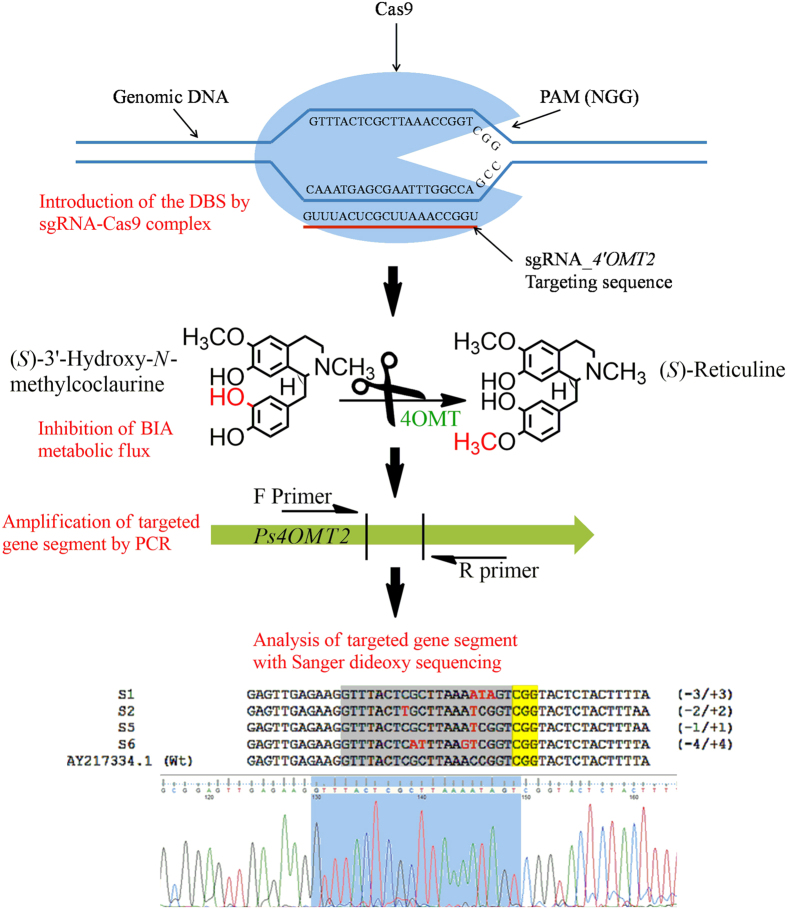
After induction of mutagenesis in the *Ps4*′*OMT* gene region by CRISPR/Cas9 system, targeted sites were amplified from outside by PCR and InDels were identified by Sanger dideoxy sequencing. Both results show that genome knockout lines were generated and the majority of gene knockout deleted 1 to 4 nt from the genome sequences.

Constructed plasmids of 46965 (pK7WGF2::hCas9), pICH47751::AtU6p::sgRNA_4OMT2, and pTRV2::AtU6p::sgRNA_4OMT2 were transformed into opium poppy by *Agrobacterium*-mediated transformation to induce the expression of sgRNA and Cas9 in leaf tissues. To identify InDels in the target region, an isolated gDNA sample was enriched by using AgeI restriction enzyme to eliminate non-targeted *4*′*OMT2* gene segments and was then amplified by PCR and subjected to Sanger sequencing. A total of 48 clones were sequenced and used for InDel analyses. InDel sequence analysis revealed that 16 out of 20 (80%) randomly sequenced clones showed deletions from the synthesized -sgRNA and 24 out of 28 (85%) clones showed deletions as a result of viral-based sgRNA-mediated endonuclease activity of Cas9 (Fig. 2). The majority of the identified InDels were located at the region targeted by the seed sequence of sgRNA. The consequential mutations ranged from 1 to 4 bp insertion/deletions in the targeted segment. These results support the idea that CRISPR/Cas9 system is a precise and reliable tool to knock-out the gene of interest in the opium poppy genome with a huge genome size of nearly 3.3 Gb^48^.

To identify the presence of any potential off-targets, we used the online BLASTN tool of NCBI. The 20-nt sgRNA sequence was used to search for off-target candidate sites in the *P. somniferum* genome^49^. We identified a single candidate gene, *4*′*OMT1* (isoform-1), which had 17 bp matches out of 20 bp and 94% identity with a single base difference 9 bp upstream of the 3′ end.

### The impact of CRISPR/Cas9 mediated gene knock out on plant BIA biosynthesis

**Figure 3:**
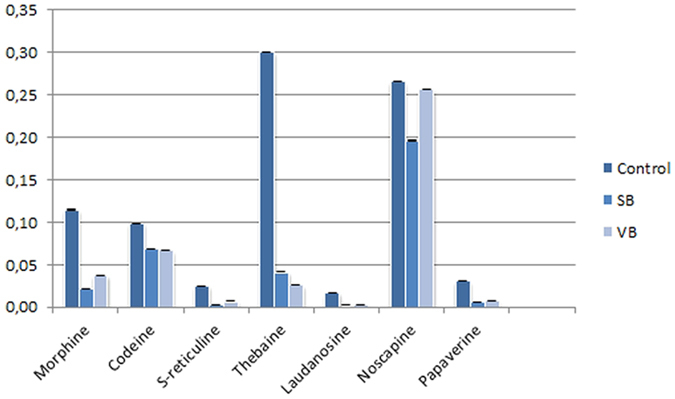
Alkaloid levels were signifncatly reduced in CRISPR/Cas9 knockout opium poppy leaves measured by HPLC-ToF/MS. The amounts were ×10^−3^ mg/g. Error bars represent SD. *P < 0.005, using one-way ANOVA Tukey’s test. SB: Synthetic Backbone; VB: Viral Backbone.

**Figure 4:**
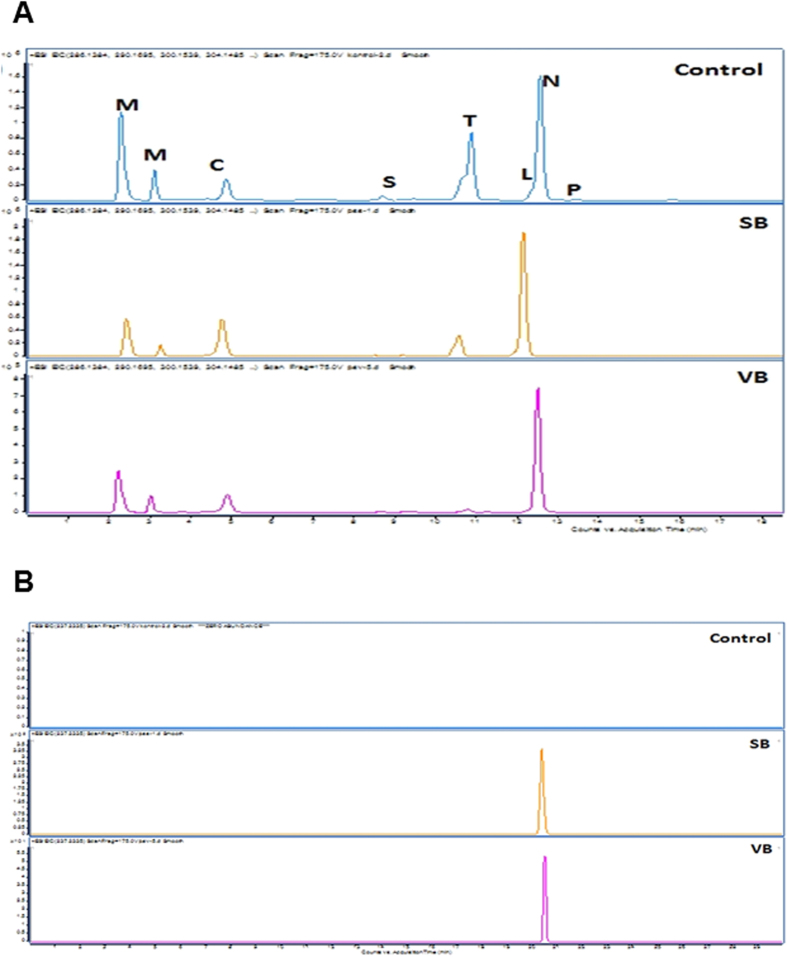
The chromatogram of the measured alkaloids in genome knockout leaves as well the control leaves. (**A**) known alkaloids; M: morphine isomers; C: codeine; S: S-reticuline; T: thebaine L: laudanosine; N: noscapine; P: papaverine, (**B**) unidentified peak only represented in CRISPRed plants.

To determine the efficiency of CRISPR/Cas9-mediated gene knockout, we employed HPLC-ToF/MS to measure the expression levels of several alkaloids (morphine, codeine, *S*-reticuline, noscapine, thebaine, laudanosine and papaverine) regulated by the *4*′*OMT2* gene. HPLC-ToF/MS data showed that the production of these detected alkaloids was significantly reduced in gene knockout plants (*P* < *0.005*) (Figs 3 and 4; Supplementary Table S2). The level of total alkaloid accumulation was reduced in both synthetic and viral-based CRISPR-knockout plants. Interestingly, the decrease of total alkaloid in synthetic-based CRISPR-mediated plant knockout was higher than viral-based applications. In addition, *S*-reticuline was significantly decreased in the opium poppy leaves of the knockout plants and the most reduced alkaloid was found to be thebaine (Fig. 3). Also, the biosynthesis of the end-products morphine, noscapine and papaverine was reduced.

## Discussion

This study demonstrates that successful application of the CRISPR/Cas9 system in opium poppy is possible by both synthetic and TRV-mediated systems. Previously, the TRV-based post-transcriptional gene silencing approach was successfully applied in opium poppy^50^,^51^. We took advantage of the efficiency of the system and used the TRV vector to express sgRNAs to improve the CRISPR/Cas9 system. TRV, as a viral plasmid, can systemically distribute sgRNAs from the transfected cell to its neighbouring plant cells. Additionally, there was a published report about the usage of TRV vectors for sgRNA transcription in Cas9 encoding tissues of *N. benthamiana*^52^.

In this study, we successfully showed the effectiveness of CRISPR/Cas9 system in *P. somniferum*, using synthetic and viral plasmid backbones to knock-out the *4*′*OMT2* gene which has a role in the regulation of the biosynthesis of many valuable medicinal aromatic metabolites including noscapine and morphine. The delivery method of the sgRNA/Cas9 system into plant cells is a limiting factor for highly efficient CRISPR/Cas9-mediated gene editing. Conventional *Agrobacterium*-mediated gene transformation requires more time to see results. With this in mind, we employed *Agrobacterium*-mediated leaf infiltration to deliver the Cas9 and sgRNAs into plant cells and investigate the gene editing efficiency. The *4*′*OMT2* gene was knocked-out using sgRNAs transcribed from both viral (TRV) and synthetic backbone vectors. No significant differences in efficiency were observed between those different applications. This could be due to using synthetic vectors for hCas9 in both strategies, which could have masked potential differences. When applying CRISPR/Cas9 in plants, it is not possible to express both Cas9 and sgRNA from a single viral vector. This is due to the low-cargo capacity of plant viral vectors. Hypothetically, some strategies could be used to overcome these obstacles. For example, the Cas9 sequence could be split and transferred into organisms. Then, it could be triggered to re-assemble into a functional protein form *in vivo*^53^. Another possibility is to use a smaller *Staphylococcus aureus* Cas9 protein which is one third the size of SpCas9 53,54. Such approaches can facilitate the expression of Cas9 endonuclease in plant tissues by using virus-based vectors. The TRV system works systemically in plant cells and it’s preferred for the delivery of sgRNA and human-codon optimized Cas9 (hCas9) coding sequences into the plant leaves^52^. It was thus used to express sgRNA_4OMT2 and sgRNA_PDS under an *AtU6* promoter.

Analysis of off-target effects with the web-based NCBI database was limited due to lack of genome sequence information for the opium poppy. The homology between *4*′*OMT2 and 4*′*OMT1* was not considered problematic due to the recognition requirements for Cas9 endonuclease activity on its targets, which is based on the presence of an upstream PAM sequence. Mismatches at the 3′ end adjacent to the PAM sequence are not tolerated by the CRISPR/Cas9 system^55^. Therefore, this system can distinguish between *4*′*OMT2* and *4*′*OMT1* despite their high homology.

We also showed that the metabolite accumulation level in opium poppy was influenced by CRISPR/Cas9 based gene-editing (Fig. 3). As expected, the total alkaloid content was reduced in the case of gene editing. The reduction in the alkaloid might due to the low production of downstream compounds of BIA pathway. Consistent with previous results, the silencing of *4*′*OMT2* resulted in a considerable decrease in thebaine, codeine, noscapine, and papaverine levels in stem^41^. Additionally, we detected a dramatic reduction in the *S*-reticuline and laudanosine content which are products of *4*′*OMT2* (Fig. 4). In accordance with our previous report, *4*′*OMT2* silencing led to the decrease of *S*-reticuline levels, indicating its possible role in morphine and noscapine biosynthesis^47^. We also detected a slight reduction in thebaine levels which is likely mediated by the RNA-based silencing of *4*′*OMT2* in leaf^47^. This might be explained by the high thebaine accumulation in control plants.

Consequently, our study demonstrated that CRISPR/Cas9 system is applicable in *P. somniferum* which can be used as a framework in future studies to address some probable questions regarding the BIA biosynthesis pathway (Fig. 1). By applying CRISPR/Cas9 in *Papaver* species, opium poppy and related species can be converted into Biofactories for the mass-production of BIAs simply by introducing breaks in related gene sequences. The CRISPR/Cas9 system provides a wide range of applications, with high accuracy and precision in comparison to other genome editing tools. Therefore, it will be a commonly used technique for plant metabolic engineering in the near future.

## Additional Information

**How to cite this article**: Alagoz, Y. *et al*. Manipulating the Biosynthesis of Bioactive Compound Alkaloids for Next-Generation Metabolic Engineering in Opium Poppy Using CRISPR-Cas 9 Genome Editing Technology. *Sci. Rep*. **6**, 30910; doi: 10.1038/srep30910 (2016).

## Supplementary Material

Supplementary Information
