# Supplementary material for: Manipulating the Biosynthesis of Bioactive Compound Alkaloids for Next-Generation Metabolic Engineering in Opium Poppy Using CRISPR-Cas 9 Genome Editing Technology
Source: Sci Rep. 2016 Aug 3;6:30910. doi: 10.1038/srep30910 (PMC4971470; doi:10.1038/srep30910)
Supplement: Supplementary Information [file srep30910-s1.pdf]

Supplementray Materials to:

Manipulating the Biosynthesis of Bioactive Compound Alkaloids for Next-Generation Metabolic Engineering in Opium Poppy Using CRISPR-Cas 9 Genome Editing Technology

Yagiz Alagoz[1,2,+], Tugba Gurkok[1,+], Baohong Zhang[3], Turgay Unver[1,4,*]

[1] Department of Biology, Faculty of Science, Çankırı Karatekin University, Çankırı18100, Turkey

[2] Hawkesbury Institute for the Environment, Western Sydney University, Richmond, New South Wales 2753, Australia

[3] Department of Biology, East Carolina University, Greenville, NC 27858 , USA

[4] Izmir International Biomedicine and Genome Institute (iBG-izmir), Dokuz Eylul University, Balcova 35340 Izmir, Turkey

Running title: Genome editing in opium poppy

[*] Corresponding author

E-mail: turgay.unver@deu.edu.tr

+ Y. Alagoz and T. Gurkok contributed equally and should be considered as co-first author

Table S1. Primers used in this study.

Primer Name	Sequence
**4OMT2_AgeIF**	TCCCAGTGCTCAGGCTATCT
**4OMT2_AgeIR**	CGGCCAGTAAAGGAAAAAGA
**4OMT2sgRNA_1F**	tgtggtctcaATTGTTTACTCGCTTAAACCGGTgttttagagctagaaatagcaag
**4OMT2sgRNA_1R**	tgtggtctcaAGCGTAATGCCAACTTTGTAC
**M13F**	GTAAAACGACGGCCAGTG
**M13R**	GGAAACAGCTATGACCATG
**flCas9F02**	CGAAGAGAACCCGATCAACG
**flCas9R03**	CCTGAGGATAGCGTGCAGTT
**sgRNApaIF**	GGGCCCTGATCAAAAGTCCCACA
**sgRNApaIR**	GGGCCCTAATGCCAACTTTGTACAAG

Table S2. The mass and the retention times of alkaloids.

Alkaloid	mass (m/z)	retention time (min)
Morphine	286,1314	3,260
Codeine	300,1539	4,830
S-reticuline	330,1638	8,440
Thebaine	312,1536	10,590
Laudanosine	358,1952	11,070
Noscapine	414,1479	12,32
Papaverine	340,149	12,080
unidentified	237,2335	20,28

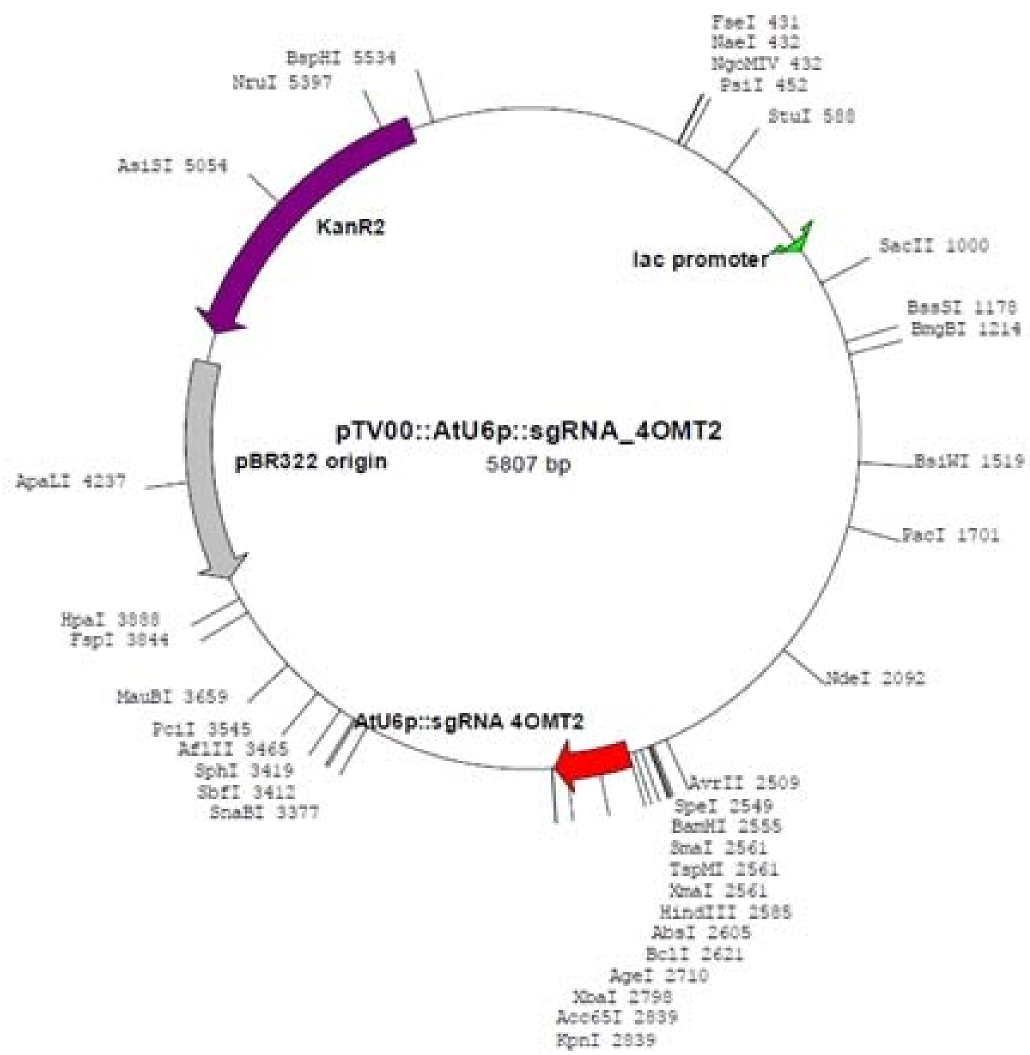

Figure S1. Map of manually designed viral based construct, pTRV2::AtU6p::sgRNA_4OMT2.

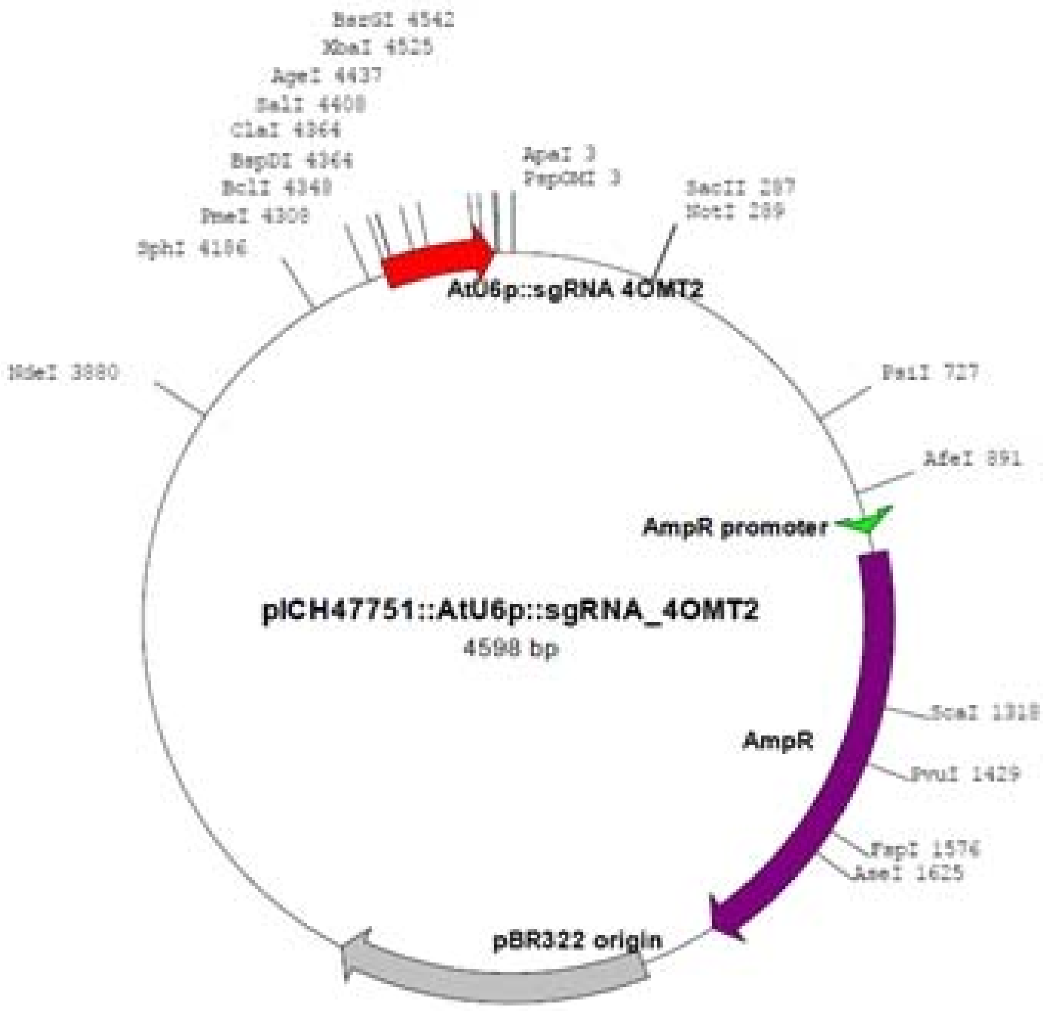

Figure S2. Map of designed synthetic construct, pICH47751::AtU6p::sgRNA_4OMT2.

a

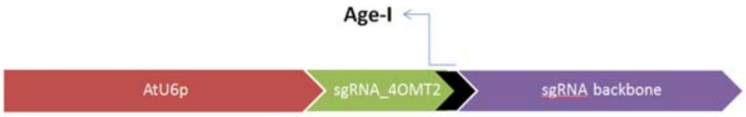

AtU6p:sgRNA_4OMT2

GGAGTGATCAAAAGTCCCACATCGATCAGGTGATATATAGCAGCTTAGTTTATATAATGATAGAG
TCGACATAGCGATTGTTTACTCGCTTAAACCGGTGTTTTAGAGCTAGAAATAGCAAGTTAAAAT
AAGGCTAGTCCGTTATCAACTTGAAAAAGTGGCACCGAGTCGGTGCTTTTTTTCTAGACCCAG
CTTTCTT**GTACAAAGTTGGCATTACGCTT**

b

```
CLUSTAL O(1.2.1) multiple sequence alignment


4'-O-methyltransferase1      ------------aaacgtagtcttcttctcaattaagaaatattctacaaacatcat---
4'-O-methyltransferase2      aaagccaaagcaaaaaactgtcttctcttcttgacaattatatacagcaaaaatcgcata
sgRNA                        ------------------------------------------------------------

4'-O-methyltransferase1      -attatgggtagta---------gtatagatgctgaaacacatgaagttgacatcaaaga
4'-O-methyltransferase2      tctaatgggtagtttagatgcaaaaccagctgctgcaacacaagaagtttccatcaaaga
sgRNA                        ------------------------------------------------------------

4'-O-methyltransferase1      tcaagctcaattatggaatataatctatggttatgctgattctcttgttcttcgttgcac
4'-O-methyltransferase2      tcaagctcaactatggaatataatctatggttttgctgattctcttgttcttcgctgcgc
sgRNA                        ------------------------------------------------------------

4'-O-methyltransferase1      agtagagattggaatcgcagatattatcaaaaacaataatgggtcaatcacactttcaga
4'-O-methyltransferase2      ggtagagattggaatcgcggatattatcaaaaacaatgatggggcaatcacacttgcaca
sgRNA                        ------------------------------------------------------------

4'-O-methyltransferase1      gcttgtatcgaaactaccactttcaaatgtgaattctgataatttgtacagacttctaag
4'-O-methyltransferase2      acttgcggcgaaactcccaattacaaatgtcagttctgattacttgtacagaatggtaag
sgRNA                        ------------------------------------------------------------

4'-O-methyltransferase1      atacttagtacacctgaatatcttaggacaacaaacttgtgctgctggagttgatagggt
4'-O-methyltransferase2      atacttggtacacctgaatatcatagaacaagaaacttgtaacggcggagttgagaaggt
sgRNA                        -------------------------------------------------------ATTGT
                                                                                    *  **

4'-O-methyltransferase1      ttactcacttaaaccagttggcacattattattaaaagattctgaaagaagcatggcacc
4'-O-methyltransferase2      ttactcgcttaaaccggtcggtactctacttttaagagatgcagaaagaagtatggtgcc
sgRNA                        TTACTCGCTTAAACCGGT------------------------------------------
                             ******.********.**

4'-O-methyltransferase1      agtaatattaggcctgtcacaaaaagatttcttgtttgtatggaatttcgtgaaagaagg
4'-O-methyltransferase2      gatgatactaggcatgactcaaaaagatttcatggtttcatggcatttcatgaaagaagg
sgRNA                        ------------------------------------------------------------
```

Figure S3. A) Schematic representation of AtU6p::sgRNA_4OMT2. Nucleotides in red demostrates the A. thaliana U6 promoter. While greens were representing the 20 bp target sequence of 4'OMT2 gene loci (black region in between green and purple demonstrated AgeI recognition site) and purple ones for tracrRNA-crRNA chimeric RNA. B) Sequence alignment of designed sgRNA with 4'OMT1 and 4'OMT2.
